# The role and mechanism of Aβ clearance dysfunction in the glymphatic system in Alzheimer’s disease comorbidity

**DOI:** 10.3389/fneur.2024.1474439

**Published:** 2024-11-25

**Authors:** Hailang Li, Qianqian Yao, Xueyan Huang, Xiaoyan Yang, Changyin Yu

**Affiliations:** Department of Neurology, Affiliated Hospital of Zunyi Medical University, Zunyi, China

**Keywords:** Alzheimer’s disease, comorbidity, glymphatic system, Aβ, AQP4, PVS

## Abstract

Alzheimer’s disease (AD) is the leading type of dementia globally, characterized by a complex pathogenesis that involves various comorbidities. An imbalance in the production and clearance of amyloid β-protein (Aβ) peptides in the brain is a key pathological mechanism of AD, with the glymphatic system playing a crucial role in Aβ clearance. Comorbidities associated with AD, such as diabetes, depression, and hypertension, not only affect Aβ production but also impair the brain’s lymphatic system. Abnormalities in the structure and function of this system further weaken Aβ clearance capabilities, and the presence of comorbidities may exacerbate this process. This paper aims to review the role and specific mechanisms of impaired Aβ clearance via the glymphatic system in the context of AD comorbidities, providing new insights for the prevention and treatment of AD. Overall, the damage to the glymphatic system primarily focuses on aquaporin-4 (AQP4) and perivascular spaces (PVS), suggesting that maintaining the health of the glymphatic system may help slow the progression of AD and its comorbidities. Additionally, given the ongoing controversies regarding the structure of the glymphatic system, this paper revisits this structure and discusses the principles and characteristics of current detection methods for the glymphatic system.

## 1 Introduction

Alzheimer’s disease (AD) is a prevalent form of dementia, accounting for approximately 60–70% of all dementia cases globally. Its pathogenesis is complex, with amyloid β-protein deposition still recognized as the central pathological feature of AD. Familial AD is associated with excessive Aβ production, whereas sporadic AD, which constitutes the vast majority of cases, is linked to dysfunction in Aβ clearance. The glymphatic system plays a crucial role in Aβ clearance, and its dysfunction leads to the reduced Aβ clearance capacity. Accumulation of Aβ results in neurodegeneration, neuroinflammation, impaired neuronal function, and ultimately leads to cognitive decline. Comorbidity plays a crucial role in the progression of AD, and reducing comorbidity can decelerate its advancement (1, 2). Certain AD comorbidities are linked to abnormal increases in Aβ (3). We conducted a PubMed search using the keywords of “multimorbidity/comorbidity/comorbidities AND Alzheimer’s diseases,” identifying 71 comorbidities related to dementia associated with AD from a Britain biological sample library of 2,128 patients. The results showed that hypertension, diabetes, cerebral small vessel disease, depression, osteoarthritis, chronic lung disease, degenerative diseases such as glaucoma and cataract were all comorbidities of AD (4). Researchers have focused on known comorbidities associated with Aβ deposition and found that some comorbidities of AD involve glymphatic system dysfunction. This dysfunction leads to a decreased capacity for Aβ clearance, which plays a critical role in the progression of AD and cannot be overlooked (Table 1). The purpose of this review is to review the comorbidities associated with Aβ accumulation in AD and to explore the accompanying glymphatic system dysfunction. We propose that dysfunction of the glymphatic system may be a significant factor contributing to Aβ accumulation. Furthermore, this finding provides a potential therapeutic avenue for reducing Aβ accumulation through intervention in glymphatic system function, thereby delaying the progression of AD and its comorbidities.

**Table 1:** The components of glymphatic system impairment associated with comorbidity.

Article	Comorbidity	Model	Key details of study/damaged components
Reference (98)	Type 2 diabetes	Rats	An increase in the number of GFAP-positive astrocytes and the loss of polarity of AQP4 protein
Reference (99)		Human	In the paraventricular nucleus (PVN) of the hypothalamus, there is an increased number of AQP4-immunoreactive (AQP4-IR) astrocytes, which are astrocytes that express a large quantity of AQP4
Reference (55)		Rats	Vascular injury and the absence of AQP4 in the perivascular region
Reference (56)		Rats	The clearance rate of cerebrospinal fluid contrast agent from the interstitial space in the hippocampus of rats is slowed by three-fold
Reference (100)		Human	The number of perivascular spaces (PVS) localized in the white matter convex central semioval region was higher in patients than in the control group
Reference (101)		Human	In the T2DM patient group, the ALPS index was decreased
Reference (97)	Depression	Mice	There is a reduction in arterial pulsatility and compliance, along with a loss of AQP4 polarization
Reference (40)		Mice	Melatonin treatment restored lymphatic system function and AQP4 polarization, improved sleep architecture, and corrected the abnormal expression of circadian rhythm proteins such as Per2, Bmal1, Clock, and Per1 in CUMS mice
Reference (59)		Human	The number of traumatic events is associated with an increase in PVS volume in patients with depression
Reference (102)		Mice	Reactive astrocyte proliferation
Reference (103)	CSVD	Human	Severe EPVS (enlarged perivascular spaces) are present in the basal ganglia and the semioval center
Reference (104)		Human	In patients with CSVD, EPVS around the lenticulostriate arteries in the basal ganglia were observed
Reference (105)		Human	EPVS in the basal ganglia
Reference (69)		Rats	PVS is significantly enlarged, with a marked reduction in the inflow and outflow functions of the lymphatic system, as well as a significant decrease in AQP4 polarity
Reference (68)	Hypertension	Mice	After an increase in blood pressure, although the arterial diameter remains unchanged, the plasticity of the arterial wall is altered, leading to increased reflux and reduced net flow within the PVS
Reference (106)		Rats	Proliferation of reactive astrocytes
Reference (70)		Human	In patients with hypertension, the ALPS index is decreased
Reference (107)		Human	Patients with hypertension exhibit extensive EPVS
Reference (73)	Chronic kidney disease	Human	EPVS in the frontal cortex and basal ganglia
Reference (72)		Human	Decreased DTI-ALPS index
Reference (108)		Human	Decreased DTI-ALPS index
Reference (77)	Epilepsy	Human	The DTI-ALPS index is significantly reduced and is lower in patients with poor response to antiepileptic drugs
Reference (109)		Human	Epilepsy patients exhibit a higher number and volume of PVS (perivascular spaces)
Reference (80)	Glaucoma	Mice	Enlarged perivascular spaces
Reference (110)		Mice	Retinal astrocytes expressing abundant AQP4 facilitate the clearance of Aβ from the retina and optic nerve
Reference (111)	INPH	Human	In patients, the density of AQP4 water channels in the end-feet of astrocytes along cortical micro vessels is significantly reduced
Reference (112)		Human	Patients with INPH have fewer enlarged perivascular spaces (EPVS) in the semioval center

## 2 Structure and function of the glymphatic system resource identification initiative

Cerebrospinal fluid (CSF) is produced in the choroid plexus of the lateral ventricles and it flows into the perivascular spaces (PVS). Driven by the AQP4 water channel expressed on the end-feet of astrocytes, the CSF mixes with interstitial fluid (ISF) in the brain’s interstitial space and is then cleared out through the PVS in what is known as the glymphatic system (5, 6). The main structures of glymphatic system include the arteriovenous vessel wall, astrocyte end-feet surrounding the vessel wall to form the PVS, astrocytes and AQP4 on its end-feet (7). The most commonly observed abnormalities in the pseudo-lymphatic system appear to be AQP4 and PVS. AQP4 dysfunction often results from a reduction in quantity, ectopic expression, and loss of polarization, leading to glymphatic system impairment (8–10). The role of PVS in the glymphatic system is particularly important, as most detection techniques and research related to the glymphatic system are associated with PVS. PVS are formed when small cerebral blood vessels penetrate from the brain surface into the brain tissue, accompanied by layers of meninges. This creates a double-layer structure around the vessels, providing a pathway for CSF to flow and participate in waste clearance. The composition of PVS varies across different brain regions; in the basal ganglia, small arteries are enveloped by two layers of meninges, with interstitial spaces located between these layers, directly connecting to the subarachnoid space. In the cortical region, arteries are typically wrapped by a single layer of meninges, which extends into the brain tissue, with glial cells covering the surrounding meninges, forming spaces around the vessels (11). PVS generally have a diameter of less than 2 mm, and those exceeding 2 mm are considered dilated enlarged perivascular spaces (EPVS) (12). EPVS can be observed in healthy individuals across all age groups, and the incidence of EPVS increases with age (13). An abnormal increase in their number and diameter can indicate pathological conditions and brain aging. Accumulation of inflammatory cells, leakage of cerebrovascular fluid into PVS, and age-related brain tissue atrophy can lead to PVS fibrosis and occlusion, resulting in impaired drainage of ISF that accumulates in PVS, causing their expansion (14–16).

**Figure 1:**
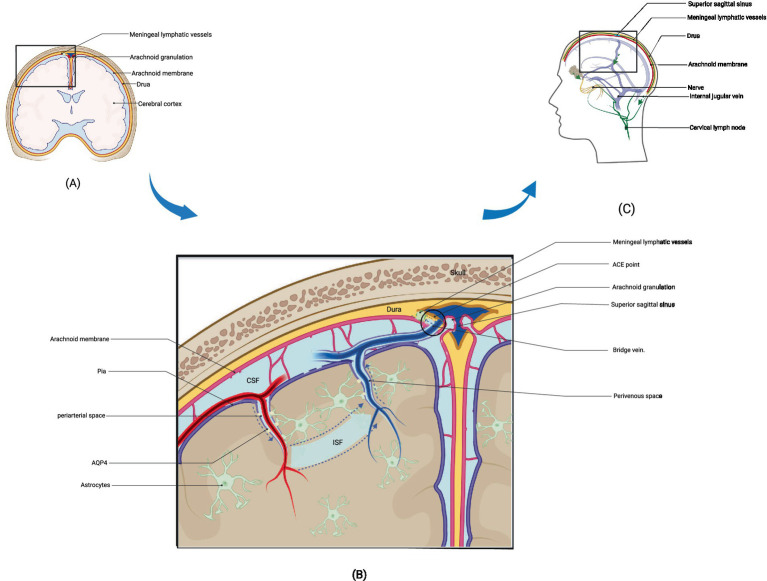
Structure and drainage schematic of the glymphatic system. (A) represents a schematic diagram of the local structure of the brain. (B) shows an enlarged view of the local structure in A, including the glymphatic system. Cerebrospinal fluid enters the interstitial space through the perivascular space around arteries under the action of AQP4, where it mixes with interstitial fluid and then leaves the interstitial space through the perivascular space around veins after mixing with metabolic waste such as Aβ. The figure illustrates the latest pathway from science regarding how cerebrospinal fluid enters the meningeal lymphatic vessels, specifically by entering the ACE point through the perivenous space and then exiting the cranial cavity via the meningeal lymphatic vessels. (C) depicts three potential pathways for drainage via the glymphatic system: drainage into cervical deep lymph nodes via meningeal lymphatic vessels, absorption into arachnoid granulations and subsequent drainage into internal jugular vein, and drainage alongside cranial nerves into cervical lymph nodes. (B) adapted from “Cranial Meninges,” by BioRender.com (2021). Retrieved from https://app.biorender.com/biorender-templates/figures/all/t-6058d9827238b100a7968023-cranial-meninges.

**Figure 2:**
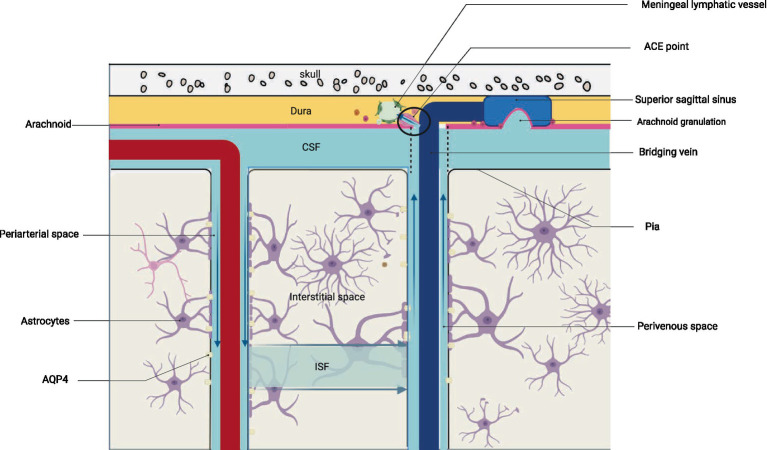
Structure and drainage schematic of the glymphatic system. The figure corresponds to Figure 1B.

Brain metabolites such as Aβ are produced and accumulated in the ISF. CSF, which mixes with ISF, is then cleared through the perivenous space. Aβ subsequently leaves the cranium by absorption through the arachnoid granules to the dural sinus and then converges into the internal jugular vein, into the cervical lymph nodes via the nerve sheath lymphatics of the cranial nerves (including the sieve plate), or by the way of the cervical lymph nodes via the foramen magnum of the occipital bone and the foramen magnum of the jugular vein and the neural foramen (17). Recent research suggests that CSF can penetrate the dura mater through the bridging veins, forming the arachnoid cuff exit (i.e., the ACE point). This fluid then enters the dura mater, where it is absorbed by meningeal lymphatic vessels and removed from the cranial cavity. After neuroinflammation, the ACE point becomes obstructed, limiting the clearance of metabolic waste products such as Aβ. The ACE point is a complex structure composed of fibroblasts from the arachnoid and dura mater, along with various immune cells (18) (Figures 1, 2).

Impaired clearance of α-synuclein by the glymphatic system accelerates Parkinson’s disease symptoms. Blockage of meningeal lymphatics due to subarachnoid hemorrhage and accumulation of neurotoxic molecules during stroke aggravate neurological damage, both of which are also associated with glymphatic system dysfunction (19). In addition, the glymphatic system affects the frequency of migraine attacks (20). And dysfunctional clearance of Aβ and Tau proteins by the glymphatic system has an important role in the development of AD (21).

## 3 Mechanisms of Aβ clearance by the glymphatic system

It is widely accepted that the movement of CSF from the PVS into the brain parenchyma interstitial space and ISF can be explained by several mechanisms. Firstly, the pulsation of cerebral arteries, combined with the contraction and relaxation of cerebral blood vessels and the pressure gradients within the CSF, facilitates lymphatic flow (7, 22, 23). Secondly, synchronized action potentials within the brain’s neural network generate large amplitude rhythmic ion waves, promoting CSF entry into the brain parenchyma for efficient clearance of metabolic waste (24). Thirdly, respiratory and cardiac vascular pulsations also influence CSF flow. During inspiration, thoracic pressure is transmitted to the subarachnoid space through the interconnected venous plexus in the spinal canal, subsequently affecting the inflow and outflow of CSF (25–27). Once entering periarterial spaces, CSF mixes with ISF, aided by AQP4, before entering perivenous spaces for eventual drainage from the brain. The removal of Aβ from the brain involves processes such as cellular phagocytosis, protease degradation, blood-brain barrier transport, and lymphatic drainage pathways. While initially believed to have a minor role in Aβ clearance, subsequent research has underscored the glymphatic system’s important role in clearing Aβ in AD patients’ brains (28). Diffusion tensor imaging along the perivascular space (DTI-ALPS) has been employed to evaluate glymphatic system function in AD patients, revealing a correlation between glymphatic system dysfunction and lower cognitive scores, thus providing imaging evidence of glymphatic damage in AD patients (29–31). Increased PVS size, alongside AQP4 gene deletion or misvocalization, affects glymphatic system functionality, leading to impaired Aβ clearance in both AD patients and experimental animal models (29, 32–34). Obstruction of glymphatic drainage by ligating the deep cervical lymph nodes can exacerbate Aβ accumulation, neuroinflammation, and cognitive impairment in AD mice. Conversely, preserving lymphatic system drainage can reduce Aβ deposition, thereby interfering with the onset and progression of AD (35). In conclusion, impaired clearance of Aβ by the glymphatic system underlies its accumulation, while enhancing Aβ elimination through the glymphatic system can slow AD development.

## 4 Sleep: a necessary condition for the normal function of the glymphatic system

During natural sleep, the brain’s interstitial space expands by 60%, indicating that the glymphatic system’s function during sleep is likely linked to this increase, resulting in heightened CSF and ISF exchange (36). Within the stages of sleep, the glymphatic system is particularly active during slow-wave sleep (SWS), evidenced by the fact that up to eight nights of partial sleep deprivation do not alter cerebrospinal fluid Aβ levels when SWS is maintained (37). When SWS is preserved, sleeping in a lateral position enhances the glymphatic system’s efficacy over prone and supine positions, facilitating the most rapid clearance of Aβ (38). Sleep-regulating hormones like melatonin play a crucial role in maintaining an optimal circadian rhythm and sleep quality, essential for the normal function of glymphatic system. Conversely, reduction in melatonin levels among the elderly correlates with an increased dementia risk (39–41). Interventions aimed at improving sleep can enhance glymphatic system functionality, highlighting the critical role of sleep in maintaining the system’s efficiency (42).

Sleep disorders result in abnormal orexin secretion, altered the expression of circadian clock, elevated neuronal activity levels, and increase in Aβ production (43, 44). Following sleep deprivation, the glymphatic system’s ability to clear Aβ from the brain diminishes (45). Imaging studies of individuals with sleep disorders and cognitive impairments reveal significantly higher PVS in certain brain regions compared to healthy individuals, correlating with elevated Aβ levels. Research also indicates that fragmented sleeps in experimental mice reduces AQP4 expression or polarization, impairing learning and memory capacities (46, 47). Moreover, genetic variations in AQP4 lead to poor sleep quality, diminished Aβ clearance by the glymphatic system, and consequent neuronal damage (48). Therefore, sleep disorders disrupt the balance between Aβ production and clearance, potentially triggering or accelerating the progression of AD in susceptible populations. Therefore, improving sleep to enhance cerebrospinal fluid flow represents a promising strategy to benefit AD patients (49–51).

## 5 AD comorbidity associated with Aβ

Comorbidity refers to the multiple chronic diseases which probably possess the same pathogenesis or different but have interaction each other, moreover, there is no complete causal relationship between the diseases (52). Comorbidities are often divided into different clusters based on disease characteristics, such as vascular-metabolic clusters and cognitive-emotional clusters (53). The incidence of AD comorbidities is increasing. Comorbidities presented before the onset of AD can promote the progress of the disease and the appearance of later complications, while comorbidities developed after AD lead to the decline of physical function and the increase of mortality. For individuals with the increasing number of comorbidities, the probability of developing AD or Mild Cognitive Impairment of AD origin will rise (3, 54). And individuals in the advanced stages of AD who also have multiple chronic diseases experience a significant decline in their quality of life (3, 4).

Based on this premise, this review explores the role and mechanisms of glymphatic system dysfunction in Aβ clearance on the background of AD comorbidities, aiming to offer novel insights for AD prevention, treatment, and delayed progression of AD.

## 6 The glymphatic system’s role in Aβ clearance with AD comorbidity

### 6.1 Type 2 diabetes

AD can be considered a central metabolic disease influenced by glucose hypometabolism. In diabetic animals, the clearance rate of contrast agent in the hippocampal interstitium was slower compared to non-diabetic rats, indicating impaired glymphatic system function. This dysfunction correlated strongly with cognitive decline, highlighting its impact on cognitive function. Glymphatic system damage includes perivascular space enlargement, astrocyte activation, and reduced perivascular AQP4 immunoreactivity (55, 56). Exosomal miRNAs play a crucial role as signaling mediators in peripheral insulin resistance target tissues and the central nervous system. The study discovered that these exosomal miRNAs can induce reduced expression and altered distribution of AQP4 in perivascular astrocytes, thereby exacerbating glymphatic system dysfunction in diabetic patients (57). Detecting glymphatic system dysfunction before cognitive impairment manifests can identify early stages of cognitive impairment earlier than immunohistopathology and cognitive testing. This is crucial for the prevention and treatment of AD.

### 6.2 Depression

Some types of depressive symptoms are associated with Aβ, potentially leading to increased Aβ deposition in the brains of depressed individuals (58). Furthermore, patients with multiple psychological trauma have glymphatic dysfunction related to the integrity of the blood-brain barrier, in which dilated PVS of the glymphatic system, confirmed that depression is a risk factor for AD, suggesting that the damage of glymphatic system may be the pathogenesis of depression leading to AD (59). In chronic unpredictable mild stress (CUMS) mice, the circulation of the glymphatic pathway is impaired and Aβ accumulation increases (60). However, administration of fluoxetine, an antidepressant medication, can reverse down-regulation and polarization of AQP4 expression, thus ameliorating deficiencies within their glymphatic system (60).

### 6.3 Cerebral small vessel disease

In the past, vascular dysfunction in comorbidities with AD has been studied as an important mechanism for the onset of AD. Vascular dysfunction can affect the level of Aβ in the brain by affecting perivascular drainage, thereby affecting the progression of AD. However, there has not been a more systematic and complete study focusing on the clearance of Aβ (61). Cerebral small vessel disease (CSVD) is a syndrome characterized by a group of small arteries, veins, and capillaries in the brain affected by various etiologies, with pathological manifestations including white matter hyperintensities and microbleeds. In CSVD, Aβ deposition is associated with impaired glymphatic clearance mediated by AQP4 and neuroinflammation mediated by microglia and astrocytes. Aβ activates microglia to release inflammatory cytokines, causing tissue damage, which in turn exacerbates Aβ deposition (62).

The glymphatic system impairment in CSVD can involve AQP4 and PVS. Loss of AQP4 polarization, reactive astrocyte formation along PVS, and damage to perivascular astrocyte end feet affect lymphatic function through blood-brain barrier damage, basement membrane thickening, and PVS expansion (63, 64). In CSVD patients, a significant reduction in the DTI-ALPS index has been observed which like AD, is directly related to cognitive function (65). Therefore, the progression of cerebral small vessel disease can be used for clinical prediction of preclinical AD or prodromal AD. There is a genetic overlap between CSVD and AD, which may form the basis for their comorbidities and glymphatic system dysfunction.

### 6.4 Hypertension

Hypertensive can also impair the glymphatic system, reducing Aβ clearance (66, 67). Research using particle tracking technology suggests that changes in arterial wall pulsation in hypertensive patients lead to reduced cerebrospinal fluid flow, increased lymphatic reflux, and decreased net cerebrospinal fluid flow in the PVS, consequently impairing glymphatic function (68). Significantly reduced influx and efflux of the lymphoid system was found in patients with essential hypertension and in animal models, with a significant enlargement of the PVS, altered AQP4 polarity, and reduced ALPS index (69, 70). This suggests that blood pressure is not only an important component of the dynamics of the glymphatic system, but also contributes to the stabilization of the PVS and AQP4 structure of the glymphatic system. Providing new insights into the coexistence of hypertension and concomitant vascular lesions in AD.

### 6.5 Other less studied comorbidities

#### 6.5.1 Chronic kidney disease

The clearance of peripheral Aβ mainly relies on the kidneys and the liver. When renal function of patients with chronic kidney disease (CKD) declines, there is an increased secondary transport of Aβ into the brain. Moreover, CKD patients demonstrate abnormal function of angiotensin-converting enzyme (an Aβ degrading enzyme), and elevated levels of cystatin C that inhibits cathepsin B in the brain, resulting in heightened Aβ accumulation (71). Among CKD patients without neurological symptoms have abnormal glymphatic systems, manifested as a decrease in the DTI-ALPS index. This may be caused by vascular damage in CKD patients leading to arteriosclerosis and diminished cerebral blood flow, accompanied by decreased expression of AQP4 (72, 73).

#### 6.5.2 Epilepsy

The interaction between AD and epilepsy is reciprocal. AD increases the risk of epileptic seizures, and there is an elevated probability of cognitive impairment in epilepsy patients. Aβ can induce synaptic damage leading to epileptic seizures, and Aβ deposition further increases following epileptic seizures (74–76). The mutual interaction mechanism between AD and epilepsy is complex, with a core involvement of Aβ alterations. Dysfunction of the glymphatic system has been observed in patients with various forms of epileptic seizures, characterized by a reduced DTI-ALPS index. For patients with poor response to antiepileptic drugs, there is often more pronounced glymphatic dysfunction, indicating that impaired glymphatic system function may exacerbate epilepsy (77–79). Enhancing glymphatic system function represents a potentially effective therapeutic strategy for managing epilepsy.

#### 6.5.3 Glaucoma

The optic nerve, as an extension of the central nervous system, has been found to have lymphatic-like drainage along the eye, allowing Aβ to be transported from the vitreous to the optic nerve for clearance through this glymphatic system. In a mouse model of glaucoma, it was observed that the expansion of PVS in the optic nerve leads to impaired glymphatic function. Additionally, aging, an independent risk factor for AD, also causes damage to the eye’s glymphatic system (80, 81).

#### 6.5.4 Idiopathic normal pressure hydrocephalus

Approximately 10% of patients with AD experience idiopathic normal pressure hydrocephalus (INPH), for ‘idiopathic normal pressure hydrocephalus both of which are characterized by astrocyte response hyperplasia and Aβ deposition at the pathological level. IPNH patients have glymphatic system damage and both have sleep disorders like AD patients, suggesting that glymphatic system damage caused by sleep disorders may be the basis for the comorbidity of the two diseases (82, 83).

## 7 Application of glymphatic system impairment in clinical settings of AD and comorbidity

Despite the extensive research on glymphatic system dysfunction, there is currently no unified clinical definition or diagnostic criteria for this condition. Given that comorbidities can arise both before and after the onset of AD, and considering that AD itself exhibits glymphatic dysfunction, distinguishing the causes of glymphatic system impairment related to comorbidities that occur post-AD is challenging. It remains difficult to determine when it is necessary to conduct evaluations of glymphatic function. Current research primarily focuses on early detection of glymphatic dysfunction in individuals with high-risk factors for AD, such as hypertension and INPH, to predict the likelihood of later developing AD (70, 82, 84). Although there are no clear clinical definitions or screening methods for glymphatic dysfunction, surgical interventions aimed at improving cervical lymphatic drainage based on the theory of glymphatic clearance impairment have been implemented in AD patients. For instance, a study by Xie et al. (85) involved reconstructing cervical lymphatics in 50 patients, resulting in significant improvements in cognitive function. Similarly, the administration of prostaglandin F2α was shown to enhance the contraction frequency of cervical lymphatics and increase cerebrospinal fluid outflow velocity in aged mice, restoring these metrics to levels seen in younger mice (86). Research on the glymphatic system in the context of AD comorbidities remains limited, with a primary focus on using the ALPS index to predict the probability of developing AD or cognitive impairment, as well as potential pathological changes such as Aβ accumulation. However, a study investigating epilepsy as a comorbidity in AD patients indicated that those with more severe glymphatic system dysfunction exhibited poorer responses to antiepileptic medications (77). This may suggest that glymphatic system impairment is associated with adverse clinical outcomes. Future research efforts should prioritize this area to better understand the implications of glymphatic dysfunction in AD and its comorbidities.

## 8 Current challenges

In experimental mice, researchers inject radioactive-labeled Aβ into the cranial cavity and use fluorescence techniques to observe a 40% impairment in Aβ clearance in aging brains (87). This study primarily relied on *ex vivo* brain slice experiments to assess clearance levels. In animal models, researchers can use drugs that are not cleared by the blood-brain barrier to specifically measure glymphatic system clearance of Aβ; however, in humans, the invasiveness of contrast agents and fluorescent dyes limits the assessment of glymphatic system function. Although some studies suggest possible measurement methods, they mainly focus on overall clearance rates rather than specifically measuring glymphatic clearance of Aβ (88). Clinically, dynamic contrast enhancement and DTI-ALPS are commonly used to evaluate glymphatic system function in patients. The DTI-ALPS technique is particularly prevalent, allowing non-invasive observation of water diffusion rates in perivascular spaces to understand glymphatic dysfunction (31). However, no study has established standard normal and abnormal ranges for the DTI index or other glymphatic measurements (89, 90). Many studies provide DTI-ALPS values relative to normal control groups, possibly due to short-term state changes affecting Aβ clearance, such as increased amyloid burden following sleep deprivation, as well as the diurnal and state-dependent variations in CSF production rate, ISF volume, ISF turnover, and glymphatic flow (91).

While the DTI-ALPS index is widely used as an indicator of glymphatic system function, its limited relevance in deep white matter regions, where CSF-ISF exchange plays a minor role, necessitates a critical evaluation of DTI technology’s focus on deep white matter and vascular distribution (92). There is an urgent need for the development and refinement of precise, non-invasive lymphatic function measurement methods. Aβ removal from the brain occurs through overlapping clearance systems, including enzymatic degradation, cellular uptake, transport across the blood–brain barrier and blood-cerebrospinal fluid barrier, and overall ISF flow to the circulation and glymphatic system. Future research should prioritize understanding the proportions of each clearance system (93, 94). Additionally, some studies suggest that dysfunction in meningeal lymphatics does not alter amyloid pathology associated with AD, indicating the need for further investigation into the relative importance of Aβ clearance pathways (95). Current research has not definitively established whether glymphatic system dysfunction is always accompanied by Aβ deposition in all comorbidities. Most studies indicate that glymphatic system dysfunction is often related to Aβ accumulation. Therefore, future research should further explore the relationship between the two or determine what proportion of patients with glymphatic system dysfunction also exhibit Aβ deposition. However, given the existing evidence of a significant correlation between these factors, strategies aimed at intervening in glymphatic system function to delay or treat AD and its comorbidities should warrant the attention of researchers.

## 9 Conclusion

Current research on the lymphatic-like system primarily focuses on PVS and AQP4. Some studies use the DTI-ALPS index to assess functional impairments in this system, although these impairments may result from PVS enlargement and changes in AQP4 status. Additionally, research on brain glymphatic system dysfunction in AD and its comorbidities lacks standardized diagnostic criteria. The DTI techniques commonly used in clinical practice also have limitations. Future studies should aim to develop more precise measurement tools or biomarkers for the glymphatic system and explore the relative importance of Aβ clearance pathways.

The clearance function of the glymphatic system in removing Aβ is impaired before Aβ deposition, suggesting that early intervention in the glymphatic system may reduce the incidence of AD and its comorbidity. In terms of treatment, AD and its comorbidity should be treated as a multi-disease system. For example, the direct improvement of drainage by polyunsaturated fatty acids or the enhancement of meningeal lymphatic vessels’ function by vascular endothelial growth factor-C can impede the progression of AD and reduce comorbidity in later stages of the disease in AD patients. These interventions have the potential to improve glymphatic drainage and ultimately benefit individuals with AD (96, 97). Multiple studies have demonstrated that sleep is essential for the normal functioning of the glymphatic system.
