# Correction: The role and mechanism of Aβ clearance dysfunction in the glymphatic system in Alzheimer's disease comorbidity

**DOI:** 10.3389/fneur.2025.1633297

**Published:** 2025-06-06

**Authors:** Hailang Li, Qianqian Yao, XueYan Huang, Xiaoyan Yang, Changyin Yu

**Affiliations:** Department of Neurology, Affiliated Hospital of Zunyi Medical University, Zunyi, China

**Keywords:** Alzheimer's disease, comorbidity, glymphatic system, Aβ, AQP4, PVS

In the published article the equal contribution statement was erroneously omitted and should be included for all authors:

‘These authors have contributed equally to this work'

The authors apologize for this error and state that this does not change the scientific conclusions of the article in any way. The original article has been updated.

